# District nurses’ use of a decision support and assessment tool to improve the quality and safety of medication use in older adults: a feasibility study

**DOI:** 10.1017/S1463423620000092

**Published:** 2020-06-04

**Authors:** Annica Lagerin, Lena Lundh, Lena Törnkvist, Johan Fastbom

**Affiliations:** 1 Senior lecturer, Department of Health Care Sciences, Ersta Sköndal Bräcke University College, Stockholm, Sweden; 2 Head of Lifestyle Unit, Academic Primary Health Care Centre, Stockholm County Council, Stockholm, Sweden; 3 Adjunct Professor of Clinical Primary Care and Nursing Care, Department of Neurobiology, Care Sciences and Society, Division of Family Medicine and Primary Care, Karolinska Institutet, Stockholm, Sweden; 4 Professor of Geriatric Pharmacology, Aging Research Center, Karolinska Institutet-Stockholm University, Stockholm, Sweden

**Keywords:** older adults, nursing care, medication management, clinical decision support systems, medication review

## Abstract

**Aim:**

To investigate whether district nurses (DNs) can identify factors related to the quality and safety of medication use among older patients via a clinical decision support system (CDSS) for medication and an instrument for assessing the safety of drug use [the Safe Medication Assessment tool (SMA)]. A secondary aim was to describe patients’ experiences of the assessment.

**Background:**

DNs in Stockholm County have the opportunity to establish special units at primary health care centers (PHCCs) for patients aged 75 years and older. The units conduct drug utilization reviews and create care plans for older adults.

**Methods:**

Nine DNs at 7 PHCCs in Stockholm County used the tools with 45 patients aged 75 years and older who used one or more drugs. Outcome measures were the number of drugs, potential drug-related problems, nursing interventions, and patient satisfaction. Prevalences of drug-related problems and nursing interventions were calculated. Eleven patients answered a telephone questionnaire on their experiences of the assessment.

**Findings:**

DNs identified factors indicative of drug-related problems, including polypharmacy (9.8 drugs per person), potential drug–drug interactions (prevalence 40%), potential adverse drug reactions (2.7 per person), and prescribers from more than two medical units (60%). DNs used several nursing interventions to improve the safety of medication use (e.g., patient education, initiating a pharmaceutical review). The patients thought it was meaningful to receive information about their drug use and important to identify potential drug-related problems. With the support of the CDSS and the SMA tool, the DNs could identify several factors related to inappropriate or unsafe medication and initiated a number of interventions to improve medication use. The patients were positive toward the assessments. Using these tools, the DNs may help promote safe medication use in older patients.

## Introduction

In Sweden, older adults’ drug use has increased significantly over the past 25 years (Craftman *et al*., 2016). Today, people aged 75 years and older in Sweden are prescribed an average of five drugs, which is a common cutoff for polypharmacy (Bergqvist *et al*., 2009). The increasing number of drugs taken by older adults is largely the result of the introduction of new drugs and treatment principles that have made it possible to treat more diseases and health problems in old age (Craftman *et al*., 2016; Lagerin *et al*., 2017; Morin *et al*., 2018). However, extensive drug use in older adults increases the risk of drug-related problems. Physiological changes due to age and disease lead to increased drug sensitivity and risk of adverse drug reactions (Bergqvist *et al*., 2009), and polypharmacy is a significant risk factor for adverse drug reactions and drug–drug interactions (Lagerin *et al*., 2017).

Over the past decade, significant improvements in older adults’ drug use have been observed in Sweden, including lower use of inappropriate drugs, inappropriate drug combinations, and several types of psychotropic drugs (Fastbom and Johnell, 2015). However, the use of various somatic drug treatments (e.g., cardiovascular and anticoagulant medications) has increased steadily, and with it, total drug use. Thus, drug use in older adults is becoming increasingly complex, and more regular reassessments and refinements are needed to achieve adequate balance between risks and benefits.

In 2016, the Stockholm County Council gave district nurses (DNs) at primary health care centers (PHCCs) the opportunity to establish special units for patients aged 75 years and older. The purpose of these Elderly Care Units is to create security for older adults and improve availability and continuity of care. Responsibilities of these units include drug utilization reviews and creating care plans for older adults.

In Sweden, DNs are specialist nurses whose responsibilities include preventing illness in the population and planning, providing, and evaluating care at PHCCs and in-home health care (Lagerin *et al*., 2014). In recent decades, DNs have spent an increasing proportion of time caring for older adults, including assessing patients’ ability to manage their medications, detecting potential drug-related problems, and communicating these potential problems to patients and general practitioners (Lagerin *et al*., 2014).

However, little is known about how DNs can help improve drug use for patients at Elderly Care Units. The present study therefore aimed to investigate whether DNs can identify factors related to the quality and safety of medication use among older patients, with the support of two clinical tools: a Swedish web-based patient-centered clinical decision support system (CDSS) for assessing quality and safety of medication in older adults, SeniorminiQ (Björkman and Lieberman-Ram, 2012), and an instrument, developed in Sweden, for assessing the safety of older patients’ drug use, the Safe Medication Assessment (SMA) tool (Gusdal *et al*., 2011). A secondary aim was to describe patients’ experiences of this assessment.

## Material and methods

### Study design and sample

This study included nine DNs working at seven PHCCs in Stockholm County. The DNs were offered a two-day course in geriatric pharmacology and the use of decision support tools to assess the quality and safety of older adults’ drug use. The course was held at the Academic Primary Health Care Centre on six occasions in 2017. A total of 54 DNs completed the course, and 10 of them agreed to participate in the study. One DN dropped out prior to baseline, as her work duties had changed; thus, nine DNs participated in the baseline assessment. Two DNs withdrew from the study after baseline because they stopped working at their original PHCC. Seven DNs were thus included in the follow-up.

The DNs included patients aged 75 years and older who visited an Elderly Care Unit and used one or more drugs. Altogether, the DNs informed 54 patients about the study during routine visits to the Elderly Care Unit and 45 agreed to participate.

### Education and support for DNs

During the course, participants learned about:

- physiological changes in aging and disease,
- older adults’ drug use and polypharmacy,
- common adverse drug reactions,
- methods for improving drug use in older adults,
- how to use the CDSS and the SMA,
- how to document nursing care interventions in accordance with the well-being–integrity–prevention–safety model (Ehnfors *et al*., 2000),
- which drugs DNs are authorized to prescribe, and
- the study protocol (verbally and in writing).

Participants also received copies of the SMA tool, including a manual explaining the purpose of each item in detail and a sheet with examples of nursing care interventions that can be used to help ensure safe medication management. Approximately two months after the course, the DNs received a two-hour follow-up session at the Academic Primary Health Care Centre. The first author (A.L.) was also available during the project to provide additional support by phone or at the workplace.

### The clinical decision support system

The DNs used a web-based, patient-centered CDSS for older adults that is freely available on the Internet, SeniorminiQ (Björkman and Lieberman-Ram, 2012). SeniorminiQ is a part of the miniQ system (Quality Pharma Medtech International AB), a Swedish CDSS for assessing the quality and safety of drug use in prescribing and drug utilization reviews in care for older adults. The CDSS is based mainly on the Swedish National Board of Health and Welfare’s ‘Indicators for good drug therapy’ for people aged 75 years and older (Fastbom and Johnell, 2015). The system has been CE marked since 2011 and therefore repeatedly tested for safety, reliability, and validity. SeniorminiQ has previously been thoroughly tested as a tool for the patients to check their drug treatment and prepare for a doctor’s visit (Björkman and Lieberman-Ram, 2012). It can be used either by patients at home or by a physician or nurse together with the patient. Based on information (entered by the user) about current drug use and symptoms, the CDSS performs an analysis with respect to quality of drug use and potential adverse drug reactions. The quality analysis includes inappropriate drugs, drug duplication, and clinically relevant drug–drug interactions. The drug–drug interactions include Class C interactions (can lead to changed effect or adverse events but can be handled with individual dosage adjustments) and Class D interactions (can lead to serious clinical consequences in the form of severe side effects or lack of efficacy, or is otherwise difficult to master with individual dosing; should be avoided).

Potential adverse drug reactions are assessed on the basis of current symptoms. These are entered in accordance with PHASE-20, a Swedish tool for estimating possible drug-related symptoms in older people. PHASE-20 was developed and tested for validity and reliability by the Drug Committee in the Uppsala Region in collaboration with R&D support, Regional Council, Uppsala Region (Hedström *et al.*, 2009). In the CDSS, a potential adverse drug reaction is defined as a symptom reported as moderate or severe by the patient and that can be linked to an adverse drug reaction classified as very common (≥1/10), common (≥1/100 to <1/10), or less common (≥1/1000 to <1/100) in the Summary of Product Characteristics for any of the drugs taken by the patient.

The program generates a printable ‘basis for discussion’ that includes questions about the quality of the patient’s drug use and potential adverse drug reactions, that is intended to empower the patient and facilitate the dialog with the patient’s physician/nurse. The printout also allows the physician/nurse to study the patient’s ‘actual’ drug list and to assess the symptoms that the patient listed in the program.

### The safe medication assessment tool

The SMA is a 20-item tool that helps DNs assess and identify factors related to unsafe medication management among older patients and make decisions about medication management. The tool has been evaluated for usefulness in primary health care (Gusdal *et al*., 2011). The evaluation indicated that the SMA could identify factors related to unsafe medication management. DNs found it satisfactorily simple, relevant, complete, and understandable. They also thought that the time required to complete it was satisfactory. The first four items are about medication management; patients are asked to describe which prescription and over-the-counter drugs they use, including routes of administration, potency, and dosage, and whether someone assists them in dispensing the drug.

The next 16 items cover domains relevant to medication safety, such as whether patients can report or show all the drugs they have been prescribed, use five or more drugs, or (in the opinion of the DN) have reduced cognitive ability/memory problems. Each item represents a potential risk factor and is assigned a number of points. The total score indicates overall medication safety. The maximum possible score is 16 points: the lower the score, the safer the medication management.

### Evaluating the quality and safety of the patients’ drug use

The DNs were instructed to ask patients who visited the Elderly Care Unit and took one or more drugs if they were interested in participating in the study. The DNs also provided patients with verbal and written information about the study and obtained written informed consent. If the patient agreed to participate, the DN, together with the patient, entered the patient’s current medications and symptoms in the CDSS and filled in the SMA. Next, the DN used the ‘basis for discussion’ from the CDSS and the outcome of the SMA assessment to review the patient’s drug treatment. If the drug treatment was inappropriate or unsafe, the DN could undertake a nursing care intervention (e.g., patient education). DNs used a questionnaire to conduct a follow-up evaluation by telephone after two months, including a new assessment using the CDSS and the SMA tool.

After the follow-up, the first author (A.L.) conducted a telephone interview with up to three patients per DNs (a total of 11 patients), asking about their experience of the medication review and subsequent interventions. The patients rated their experience on a Likert scale. Response alternatives varied by question (see Table 5). A.L. recorded the telephone interviews and took notes. The average interview took 15 min (range 10–20 min); all were conducted between August and December 2018.

### Data analysis

Data were analyzed using STATA statistical software version 14.2. Descriptive statistics were presented as numbers and proportions (%). The analyses included only those patients with complete CDSS and SMA data (*n* = 45). The interviews were analyzed with qualitative content analysis (Elo and Kyngas, 2008). Data analysis began with reading all text repeatedly to achieve a sense of the whole. Notes from each interview were then sorted into content areas. Information concerning the participants’ experiences of the medication review and subsequent interventions was identified and sorted into text units (Elo and Kyngas, 2008).

## Results

### Characteristics of the older patients

Sixty percent of the 45 participating patients were women. Men’s mean age was 82 (range 74–96) years and women’s mean age was 86 (range 78–95) years (Table 1).

**Table 1.** Characteristics of the study population (*n* = 45)

	*n*	%	Mean (range)
Age			
Men	18	40.0	81.6 (74–96)
Women	27	60.0	85.8 (78–95)
Medications			
Men	18	40.0	8.6 (4–15)
Women	27	60.0	10.0 (4–21)
SMA score			
Men	18	40.0	3.8 (1–6)
Women	27	60.0	4.2 (0–10)

SMA = Safe Medication Assessment tool.

### Assessments using the CDSS

Table 2 shows the prevalence of some measures of polypharmacy, prescribing quality, and potential adverse drug reactions. The mean number of drugs was 9.8 (range 4–21) and levels of polypharmacy (use of ≥5 drugs) and excessive polypharmacy (use of ≥10 drugs) were high. The studied measures of potentially inappropriate drug use were low with the exception of Class C drug–drug interactions, which occurred in 18 (40%) patients.

**Table 2.** Prevalence of some measures of polypharmacy, prescribing quality, and potential adverse drug reactions (*n* = 45)

Measure	Prevalence
Number of drugs [mean (range)]	9.8 (4–21)
Five or more drugs [*n* (%)]	42 (93.3)
Ten or more drugs [*n* (%)]	20 (44.4)
Inappropriate drugs [*n* (%)]	2 (4.4)
Drug duplication [*n* (%)]	1 (2.2)
Drug–drug interactions, Class C [*n* (%)]	18 (40)
Drug–drug interactions, Class D [*n* (%)]	0 (0)
Potential adverse drug reactions [mean (range)]	2.7 (0–12)

Forty of the 45 patients had at least one potential adverse drug reaction. Patients had between 0 and 12 (mean 2.7) potential adverse drug reactions. The symptoms most commonly involved in the potential adverse drug reactions were tired/exhausted (18 patients), dizzy/unsteady/high risk of falls (18 patients), itching/rash (11 patients), and dry mouth (10 patients). The drugs most commonly involved belonged to the main ATC groups’ cardiovascular system (197 out of 312 drugs), nervous system (58 drugs), and alimentary tract and metabolism (21 drugs). The top six substances were metoprolol (45 drugs), amlodipine (35 drugs), zopiclone (24 drugs), bisoprolol (19 drugs), atorvastatin (18 drugs), and omeprazole (15 drugs).

### Assessments using the SMA tool

Most of the 45 who completed the SMA item asking which drugs they used, could provide a description that matched the list in their patient records (*n* = 32; 71%). Two (4.4%) received assistance from health care professionals or pharmacists with dispensing their drugs (e.g., into a dose dispenser) and one (2.2 %) received such assistance from relatives. None had any assistance with taking their drugs from a container or dose dispenser.

The two most common factors related to unsafe medication management identified by the DNs via the SMA were the use of five or more drugs (*n* = 42; 93%) and the presence of symptoms that, in the opinion of the DN, could be indicative of adverse drug reactions (*n* = 38; 84%) (Table 3). Moreover, the DNs assessed that more than a third (*n* = 18; 40%) had a risk for drug–drug interactions. The most common symptoms of potential adverse drug reactions were dry mouth (*n* = 18; 40%), shortness of breath (*n* = 17; 38%), and dizziness/unsteadiness (*n* = 16; 36%). Eleven (24%) deliberately took a dose other than the one prescribed because they experienced reduced symptoms (*n* = 3; 6.7%), found that the drug had no effect at all (*n* = 2; 4.4%), thought it had a disruptive effect on their daily routine (*n* = 2; 4.4%), lacked financial resources (*n* = 1; 2.2%), or for another reason (*n* = 3; 6.7%).

**Table 3.** Responses to individual items on the Safe Medication Assessment tool (*n* = 45)

Items	Baseline [*n* (%)]
The patient uses five or more drugs, including drugs to be taken as needed.	42 (93.3)
The patient has symptoms that could possibly be an adverse drug effect/adverse drug effects	38 (84.4)
The patient has prescribers from more than two medical units	27 (60.0)
The district nurse suspects that there is a risk for interactions between the patient’s drugs, including drugs to be taken as needed	18 (40.0)
The patient thinks he or she has too many drugs	11 (24.4)
Without consulting with the prescriber, the patient has consciously chosen not to take his or her drug or drugs as prescribed (e.g., sometimes takes a dose other than the one prescribed or no dose at all)	11 (24.4)
The patient has difficulty explaining which disease each of his or her drugs is used for	7 (15.6)
The patient has difficulty reporting or showing all the drugs he or she has been prescribed, including drugs to be taken as needed	6 (13.3)
The patient has a complex regimen (e.g., takes drugs more than 3 times a day, takes more than 12 doses a day, is on a tapering or cyclical schedule)	6 (13.3)
The patient has difficulty swallowing his or her drugs	6 (13.3)
The patient has difficulty reporting or showing the dosage form and dosage of each drug	4 (8.9)
In the opinion of the district nurse, the patient has reduced cognitive ability/memory problems	4 (8.9)
The patient has no method or routine for remembering to take his or her drugs	2 (4.4)
The patient does not store his or her drugs adequately	2 (4.4)
The patient has difficulty understanding Swedish	1 (2.2)
In the opinion of the district nurse, the patient has alcohol-related problems	0

### Nursing care interventions

Most participating patients (89%) received at least one nursing intervention to improve the safety and quality of their medication use (Table 4). The most common interventions were information and education. The DNs booked a new visit to the Elderly Care Unit for 40% of the patients (*n* = 18) and initialized a pharmaceutical review with the doctor for over a fourth (*n* = 12).

**Table 4.** Nursing care interventions in the older patients by key words from the well-being, integrity, prevention, and safety (VIPS) model (*n* = 45)

VIPS keywords	*n*	%
Information/education about	40	88.9
What disease each drug is used for	33	73.3
If the symptoms could possibly be an adverse drug effect/adverse drug effects	27	60.0
Self-care advice (e.g., physical activity, diet, and balance training)	30	66.7
The form of administration and dosing of each drug	19	42.2
Daily routines for remembering to take drugs	17	37.8
Preventive advice on the risk of falls	15	33.3
Non-pharmacological treatment	10	22.2
Which pills can be cut and/or crushed	8	17.8
How to easily swallow pills	8	17.8
Medication management aids (e.g., a dose dispenser)	8	17.8
Proper storage of drugs	8	17.8
Other (e.g., pain advice, nutrition advice)	6	13.3
Risks of combining the prescribed drugs with alcohol	3	6.7
Complex drug treatment	1	2.2
Coordination	35	77.8
Arrange a GP appointment	18	40.0
Consult the GP (e.g., about a prescription, tiredness, and blood sugar levels)	10	22.2
Initialize a clinical medication review with a GP	12	26.7
Recommend that the patient contact their GP	5	11.1
Other (e.g., care planning, referral to a dietician, and referral to emergency care)	3	6.7
Coordinate contact between physicians regarding prescribed drugs	1	2.2
Recommend contact with a case manager or home help services	1	2.2
Follow-up via:		
Contacting the patient by telephone	22	48.9
Booking a new appointment at the Elderly Care Unit	18	40.0
Other (e.g., send patient to a diabetes nurse for follow-up or to someone who can provide help with smoking cessation)	5	11.1
Change a prescription	1	2.2
Support	25	55.6
Nursing care conversation about experiences of medication management	21	46.7
Nursing care conversation with a patients who thinks he or she has too many drugs	10	22.2
Other kinds of support (e.g., for grief)	4	8.9
Conversations about alcohol habits	0	0.0
Medication management	7	15.6
Other (e.g., advice about self-care and advice about prescriptions)	5	11.1
Offer help with putting doses into pill box	2	4.4
Offer multidose packaging (*APO-dos*)	1	2.2
Prescription	3	6.7
Of a drug by the district nurse (e.g., miconazole cream)	2	4.4
Other prescriptions by the district nurse (e.g., for incontinence aids)	2	4.4
Special care	3	6.7
Other (e.g., a referral for a microalbumin urine test)	3	6.7
Consult with GP about testing for memory problems with the Swedish version of the Mini-Mental State Examination	0	0.0

### Follow-up evaluation

At follow-up, 30 patients were assessed with SeniorminiQ and 28 completed the SMA tool. A comparison between the drug lists recorded in the CDSS at first evaluation and follow-up revealed changes in drug prescriptions in 20 patients (67%; mean 1.5 per patient): 13 discontinuations of drugs, 14 new drug prescriptions, 11 dose reductions, and 7 dose increases. The net effect of these changes was a slight increase in the mean number of drugs from 9.1 to 9.2 and in the number of patients having 10 or more drugs from 10 (33%) to 11 (37%). The prevalence of inappropriate drugs (*n* = 1; 3.3%), drug duplications (*n* = 0), and Class C drug–drug interactions (*n* = 13; 29%) was unchanged.

Most of the patients assessed with SMA at follow-up (*n* = 21; 75%) could provide a description of the drugs they used that matched the list in their patient records. Three (11%) received assistance from health care professionals or pharmacists with dispensing their drugs, two (7.1 %) received such assistance from relatives, and two (7.1 %) had assistance with taking their drugs from a container or dose dispenser.

The responses of the 28 patients who completed the SMA tool at follow-up indicated that polypharmacy and the potential for related problems were similar to that reported at baseline. For instance, nearly the same percentage as at baseline used five or more drugs (*n* = 25; 89%), had a suspected risk of drug–drug interactions (*n* = 12, 43%), and in the opinion of the DN, had reduced cognitive ability/memory problems (*n* = 2; 7.1%). There were some changes in the prevalence of potential unsafe medication management. The percentage of patients who deliberately took a dose other than the one they were prescribed without consulting the prescriber decreased from baseline (*n* = 11; 24%) to follow-up (*n* = 4; 14%) (follow-up data not shown in tables).

At follow-up, the DNs conducted additional interventions for more than three out of four patients (82%). These interventions included information and education (for 43%), coordination (for 71%), and arranging a new visit to the Elderly Care Unit (for 46%) (data not shown).

### Patients’ experiences

All 11 patients interviewed by A.L. after follow-up rated the DN’s review of their drug treatment using the CDSS and SMA tools as good or very good. The patients felt it was meaningful to receive information about their drug use and to follow up their drug treatment and its effects. All patients said they would like to have a new drug review with their DN if, for example, they received a new prescription (Table 5).

**Table 5.** Responses to questions 11 older patients were asked about their experiences of the SeniorminiQ clinical decision support system and the Safe Medication Assessment tool (SMA)

Questions	Response alternatives				
1. Do you think that SeniorminiQ seems to be a good program for checking whether your drug therapy is right for you?	Very good	Good	Somewhat good	Not good at all	Do not know
	**2**	**9**	**0**	**0**	**0**
2. The program performed a quality check and generated a number of questions for discussion as a result. Did you understand what the discussion questions were about?	Understood everything	Understood most things	Understood a little	Did not understand anything	Do not know
	**0**	**6**	**4**	**0**	**1**
3. Was there anything in the discussion questions that made you worried?	Yes		No		
	**0**		**11**		
4. Do you think that the SMA seems to be a good questionnaire for following up the safety of your medication use?	Very good	Good	Somewhat good	Not good at all	Do not know
	**1**	**8**	**0**	**0**	**2**
5. Was there any question that made you worried?	Yes		No		
	**0**		**11**		
6. After going through your medications with the district nurse, did you receive an appointment with the GP to discuss your drug therapy (a so-called clinical medication review)?	Yes		No		Do not remember
	**3**		**7**		**1**
7. Was there any change in your drug therapy or were any other actions taken? (Multiple alternatives may be selected.)	No, no changes	Yes, changed dosage of one or more drugs	Yes, one or more drugs were changed to another drug or drugs	Yes, I was prescribed one or more new drugs	Yes, other actions (e.g., a referral, additional tests)
	**10**	**0**	**0**	**1**	**0**
8. If you were given the chance, would you like to review your drug therapy with a district nurse in the same way at a new visit at the Elderly Care Unit, for example, if you are prescribed a new medicine?	Yes		No		
	**10**		**0**		

## Discussion

The most common factors identified by DNs that were related to the safety and quality of medication use were polypharmacy and symptoms that could indicate adverse drug reactions. Furthermore, in the DNs’ opinion, more than a third of the patients were at risk for drug–drug interactions at both baseline and follow-up. These results were confirmed by the analysis in the CDSS and highlight older adults’ need and desire for regular follow-ups of their drug treatment.

All the participating patients had multiple medications. The mean number of drugs was as high as 9.8 per person, and over 40% of the patients had 10 or more drugs, a common criterion for excessive polypharmacy (Hovstadius *et al*., 2010). Many older adults have multimorbidity, that is, two or more long-term health conditions (Barnett *et al*., 2012). This can lead to complex medication regimens and treatment conflicts (Hughes *et al*., 2013), in part because guidelines cover treatment for individual rather than multiple concurrent disorders.

At baseline, a quarter of the patients deliberately took a dose other than the one prescribed. Several studies on older adults’ adherence to drug treatment show adherence rates of 30–50% (Haynes *et al*., 2008; Banning, 2009). The complex factors behind such nonadherence can include sociodemographic characteristics (Mc Namara *et al*., 2017), polypharmacy (Mc Namara *et al*., 2017) and its adverse consequences (Burgers *et al*., 2010), multimorbidity (Hughes, 2004), undiagnosed dementia (Banning, 2009), alcohol problems (Cooper *et al*., 2005), depression (Cooper *et al*., 2005), complex medication regimes (Donnan *et al*., 2002), and a poor relationship with health care professionals and/or care organizations (Vik *et al*., 2004; Henriques *et al*., 2012). Another contributing factor could be the feeling of having too many medications. At baseline, a fourth of all participants in the current study thought they had too many drugs.

A striking finding was that at baseline, DNs intervened with nearly all participants to improve medication use, and at follow-up with more than three-quarters. At baseline, the main interventions were information and education. This need for education is in accordance with the findings of an earlier study, which indicated that more than 40% of 75-year-olds who received a preventive home visits from a DN said they lacked knowledge and understanding about their own health problems (Sherman *et al*., 2012). It is possible that a lack of understanding of one’s health problems and of the help that medication can provide, may have contributed to self-reported nonadherence, which declined between baseline and follow-up. Patient education is a traditional approach to promoting adherence to medication in older adults (Banning, 2009), and earlier studies have found that effective information and education can lead to better adherence (Bastiaens *et al*., 2007; Moen *et al*., 2009).

Other interventions provided by DNs included helping patients with methods for remembering to take drugs, providing advice on preventing falls, and coordinating appointments (e.g., with GPs for medication reviews). Many patients received multiple interventions, such as information and a GP appointment. Previous studies show that interventions involving more than one technique can increase medication adherence by up to 41% (Schroeder *et al*., 2004).

The SMA was also designed to explore potential cognitive problems in respondents, as reduced cognitive ability can lead to problems with the quality and safety of medication management (Gusdal *et al*., 2011; Mc Namara *et al*., 2017). At both baseline and follow-up, the DNs judged that approximately 8% of the participating patients might have had cognitive difficulties. However, we do not know how many of these potentially at-risk patients the DNs would have identified without the SMA.

As a result of the evaluations using the CDSS and SMA, a high number of GP contacts were initialized. The DNs arranged a GP appointment for 40%, consulted the GP for 22%, and initialized a medication review for 27% of the patients. A comparison between drug lists in the CDSS at first evaluation and follow-up showed that changes in drug prescriptions were made for the majority (67%) of patients. However, the net effect of these changes on the extent and quality of drug use was negligible. A likely explanation for this finding is that both over and under treatment were identified.

DNs working at Elderly Care Units are in a favorable position to identify and act upon potential problems with the quality and safety of drug use in older adults. The structured assessment in the present study, using both a CDSS and SMA, provided a basis for discussion with patients and for communication with prescribers. It enabled DNs to identify patients in need of different kinds of interventions, ranging from education to further appointments with other health care professionals. Our results also indicate an apparent need for regular follow-up, as DNs identified a need for intervention in 9 of 10 patients at baseline and in 3 out of 4 at follow-up. Moreover, patients expressed the wish for a new, similar drug review with their DN if, for example, they received a new prescription.

The main limitation of this study was the relatively small number of DNs and patients who participated. The Elderly Care Units are a relatively new addition to PHCCs, so they are still under development, and some experience high personnel turnover. In addition, approximately half the patients who responded at baseline either declined to participate at follow-up or were excluded from the follow-up analyses because of incomplete responses. The low number of participants at follow-up made it difficult to draw conclusions about changes possibly caused by the interventions.

We reason that the relatively high number of incomplete responses may reflect DNs’ high workloads, which make any additional tasks burdensome. The DNs reported that completing all of the study tasks, including using the CDSS and SMA and deciding on and documenting nursing care interventions, took about an hour per patient.

## Conclusion

With the support of the CDSS and the SMA, the DNs could identify several factors related to inappropriate or unsafe medication, and the patients were positive toward the assessments. Thus, this method may be useful in promoting better and safer medication use in older patients.

Polypharmacy and adverse drug reactions were common, and at both baseline and follow-up, DNs needed to intervene to improve medication use for the majority of patients. These findings underscore the importance of DNs’ role in regularly following up older patients’ drug treatment in collaboration with other health care professionals.
